# Morphology of gracilis muscle and the topographic anatomy of its neurovascular pedicles: A cross sectional study

**DOI:** 10.12688/f1000research.144786.1

**Published:** 2024-04-18

**Authors:** Chettiar Ganesh Kumar, Rajanigandha Vadgaonkar, M.D. Prameela, Vandana Blossom, B.V. Murlimanju, Mamatha Tonse, Mangala M. Pai

**Affiliations:** 1 Department of Anatomy, Kasturba Medical College, Mangalore, Manipal Academy of Higher Education, Manipal, Karnataka, India

**Keywords:** Gracilis Muscle Flap; Pedicled Flap; Reconstructive Surgical Procedures

## Abstract

**Background:**

The objective of this study was to perform morphometry of the gracilis muscle and understand the topographical basis of the entry of its pedicles.

**Methods:**

We studied forty-four cadaveric lower extremities fixed in formalin. The length and width of the gracilis were measured at three locations: the origin, midpoint, and musculotendinous junction. The topographic location of the gracilis pedicles was also studied.

**Results:**

The gracilis muscle’s length was 369.9±34.1 mm and 359.6±29.6 mm over the right and left sides. The width of right gracilis was 25±8.4 mm, 20.4±6.2 mm and 10.6±5.6 mm at the origin, midpoint and at the musculotendinous junction. The same over the left side were 26.7±8.6 mm, 20.6±9.1 mm and 10.4±6.4 mm respectively. The number of gracilis ranged from one to three. The location of first pedicle was 93.6±35.6 mm and 68.9±35.8 mm away from the pubic tubercle on the right and left sides, respectively. The second and third pedicles were entering at a distance of 153.1±38.8 mm and 101.3±20.8 mm, 214.6±86.8 mm and 145.3±124.4 mm over the right side and left side. The accessory head of the origin of the gracilis was observed in only one cadaver (2.3%), which originated from the adductor longus.

**Conclusions:**

It is believed that the morphological data of the gracilis and its neurovascular pedicles will be enlightening to the operating surgeon. They will guide the procedures for reconstructive plastic surgery.

## Introduction

Gracilis is often utilized as a graft in the procedures of reconstruction and functioning muscle transfer. ^1^ It is excellent for the closure of rectovaginal and rectourethral fistulas. The distal pedicle is used during total knee arthroplasty as a soft tissue graft. ^2^^,^^3^ It also has wider application in the management of wounds of the groin, contractures, scars, reanimation of the face, ^4^^,^^5^ reconstruction of the mammary gland, ^6^ and anterior cruciate ligament reconstruction. ^7^ Additional harvesting of the gracilis has proven to offer better postoperative functional activity, such as in hamstring injury. ^8^ The main vascular pedicle of the gracilis is from the medial and lateral circumflex femoral arteries, which branch from the deep femoral artery, which enters the gracilis at the junction of its upper and middle third. The distal part of the gracilis is supplied by the femoral artery. ^9^ After the literature search, we found few studies on the dimensions of the gracilis muscle and topography of the neurovascular pedicle in the Indian population. The goal of this anatomical study was to determine the morphometry of the gracilis and the topography of its pedicles.

## Methods

**Figure 1.**
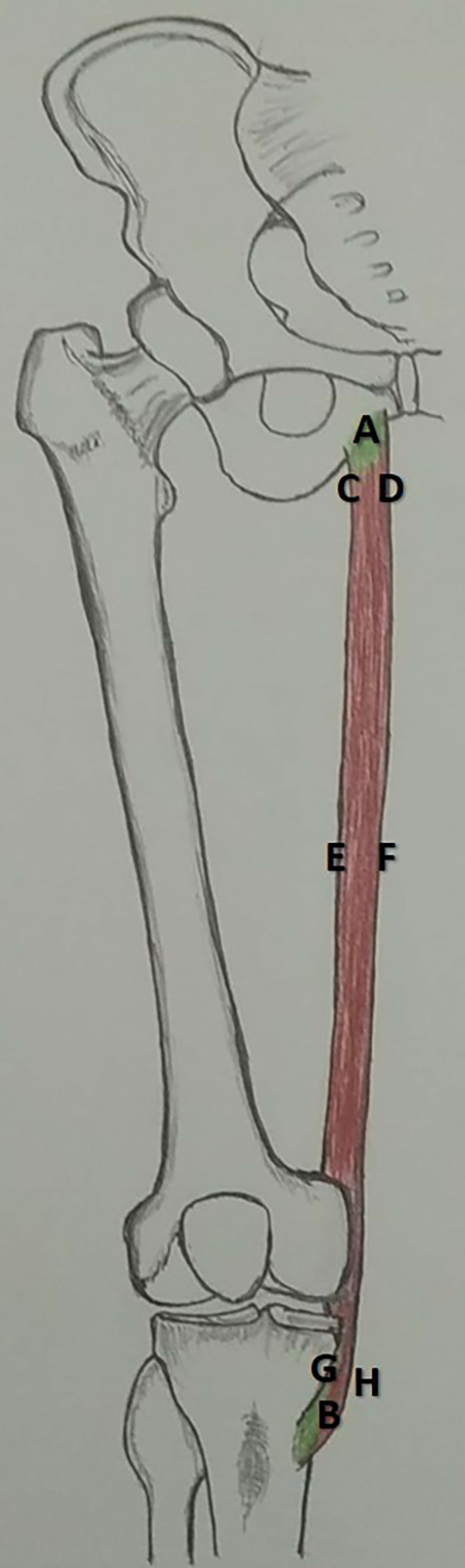
Schematic representation of the dimensions measured in this study (AB-length of gracilis, CD-width at origin, EF-width at midpoint, GH-width at musculocutaneous junction).

This study involved formalin-fixed lower-limb specimens that were available at the Department of Anatomy. A total of 44 specimens were studied, of which 25 were right-sided and 19 left-sided. Sex was not considered. Specimens with congenital malformations were excluded from this study. The institutional ethics committee, Kasturba Medical College, Mangalore (Reg. No. ECR/541/Inst/KA/2014/RR-17) has reviewed the protocol of this study and approved on seventeenth of October, 2018 (IEC KMC MLR 10-18/395). After the meticulous dissection of the adductor compartment of the thigh, gracilis muscle was exposed. This was cleaned from its origin to the dissection. The length of the gracilis (AB in Figure 1) was measured from its origin to the musculotendinous junction. The width was measured at three different points: the origin (CD in Figure 1), midpoint (EF in Figure 1), and musculotendinous junction (GH in Figure 1). The pedicles of the gracilis were counted, and their distance from the pubic tubercle was measured. The length and width were measured using a measuring tape and digital Vernier caliper (Mitutoyo, Japan), respectively. The topographical location of the pedicle entry was measured using a measuring tape. Each dimension was measured three times by three different persons, and the average was considered to avoid intra-observer bias. SPSS software, https://www.ibm.com/products/spss-statistics (version 27) was used to perform the paired t-test. We confirm that, our institution has obtained a copyright license of this software. The protocol of this study was archived in the dx.doi.org/10.17504/protocols.io.e6nvwdm6dlmk/v1.

## Results

**Figure 2.**
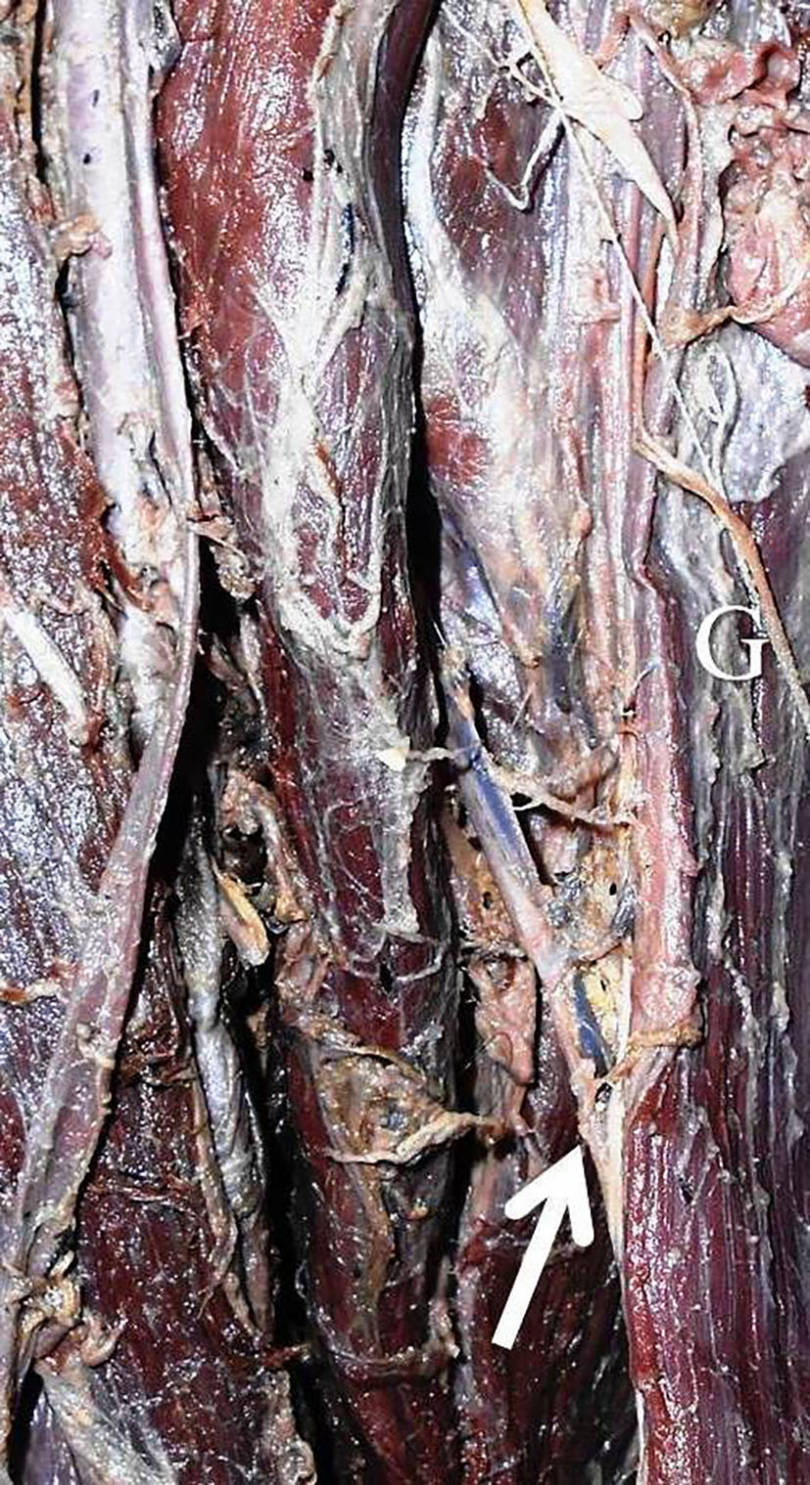
Right lower extremity of the embalmed cadaver showing the single neurovascular pedicle (indicated by arrow) of the gracilis.

**Figure 3.**
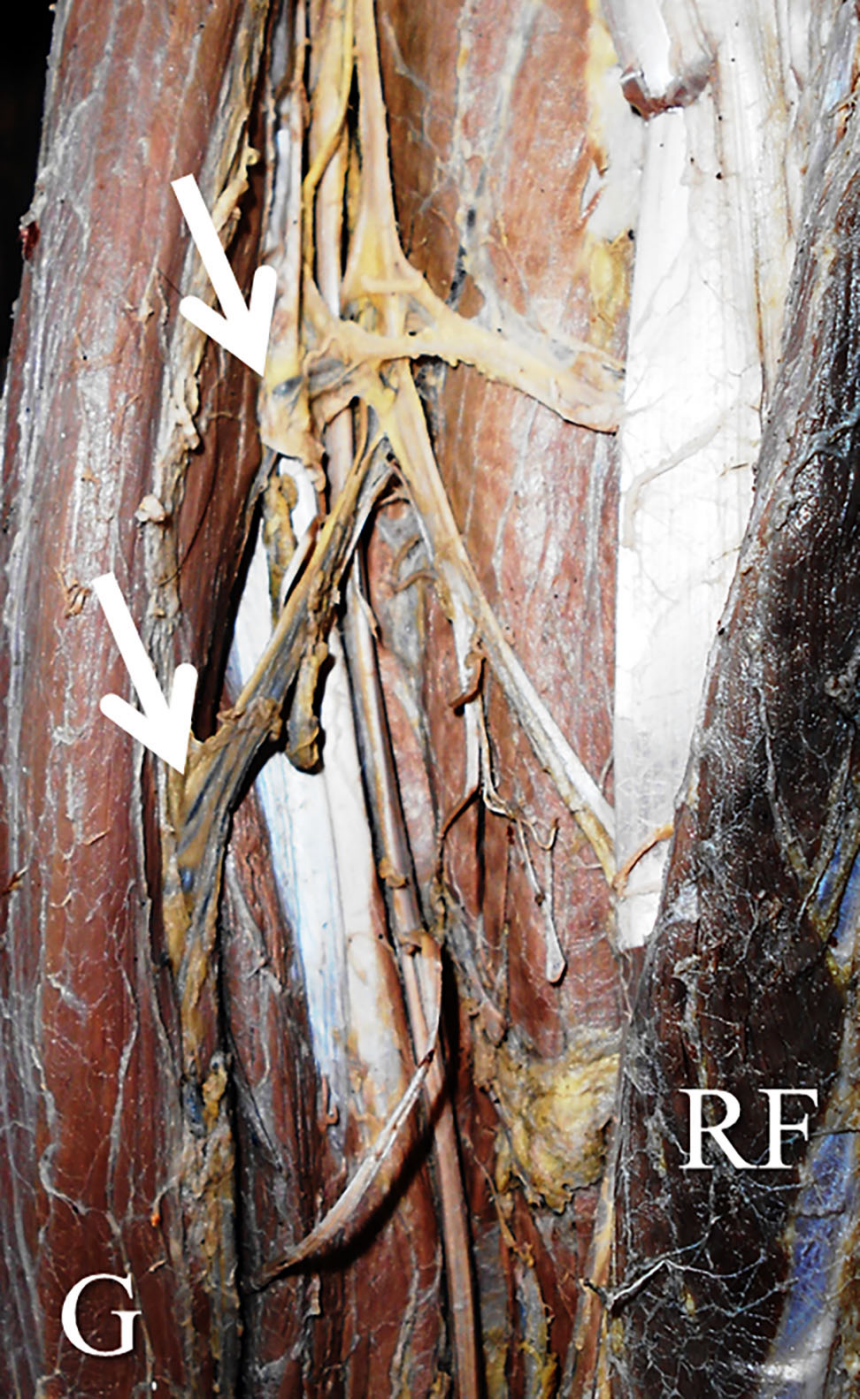
Left lower extremity of the embalmed cadaver showing the two neurovascular pedicles (indicated by the arrows) entering the gracilis.

**Figure 4.**
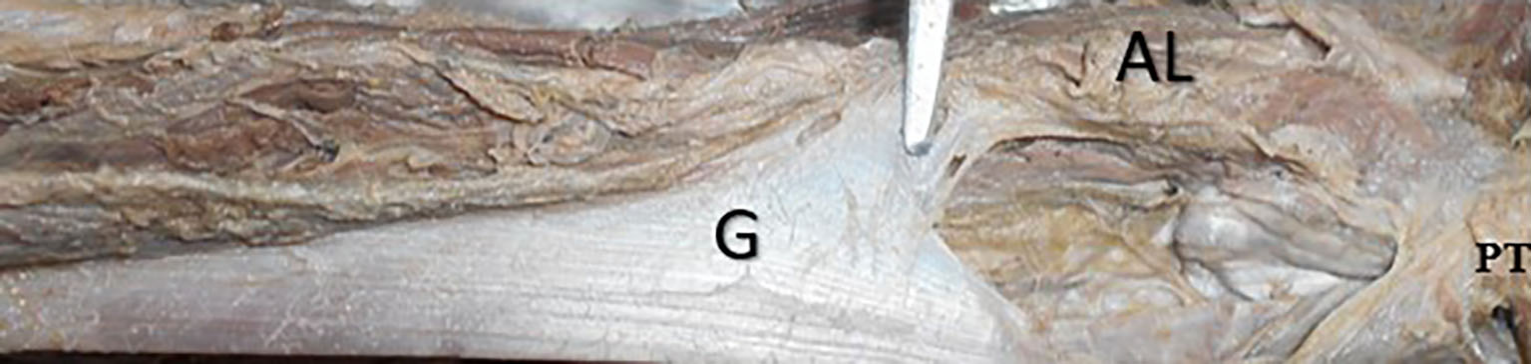
Cadaveric specimen showing the accessory head of origin of gracilis (2.3%) from the adductor longus muscle.

The length of right and left gracilis were 369.9±34.1 mm and 359.6±29.6 mm ( Table 1). The width of right gracilis was 25±8.4 mm, 20.4±6.2 mm and 10.6±5.6 mm at the origin, midpoint and at the musculotendinous junction. The same over the left side were 26.7±8.6 mm, 20.6±9.1 mm and 10.4±6.4 mm respectively ( Table 1). There were no statistically significant differences in this side-based comparison (p>0.05, paired t-test). The number of pedicles ranged from one to three ( Figures 2 and 3). The first pedicle was 93.6±35.6 mm and 68.9±35.8 mm away from the pubic tubercle on the right and left side. The second and third pedicles were entering at a distance of 153.1±38.8 mm and 101.3±20.8 mm, 214.6±86.8 mm and 145.3±124.4 mm over the right side and left side ( Table 2). The left gracilis had a proximal second pedicle compared to the right side (p<0.05). The accessory head of origin of the gracilis was observed in only one cadaver (2.3%), which originated from the adductor longus ( Figure 4).

**Table 1.** Side based comparison of measurements of gracilis muscle (n=44).

Dimension	Right side (n=25)	Left side (n=19)
Length	369.9±34.1	359.6±29.6
Width at its origin	25±8.4	26.7±8.6
Width at midpoint	20.4±6.2	20.6±9.1
Width at musculotendinous junction	10.6±5.6	10.4±6.4

Measurement in mm, paired ‘t’ test.

* Significance, p>0.05.

**Table 2.** Topographic distance of entrance of neurovascular pedicles of gracilis from its origin (n=44).

Pedicle	Right side (n=25)	Left side (n=19)
First	93.6±35.6	68.9±35.8
Second *	153.1±38.8	101.3±20.8
Third	214.6±86.8	145.3±124.4

Measurement in mm, paired ‘t’ test.

* Significance, p>0.05.

## Discussion

In the total knee arthroplasty, one of the complications is the poor wound healing and cutaneous necrosis, which requires thin pliable tough flaps. These soft tissue defects pose a challenge for filling and pedicling the medial head of the gastrocnemius flap. However, in cases where the gastrocnemius flap is insufficient, the surgeon can opt for a distally placed gracilis muscle flap. ^10^^,^^11^ Although the proximal pedicle of the gracilis is extensively utilized in plastic and reconstructive surgeries, distally placed pedicled gracilis flaps are another alternative. The distal pedicle is simple and requires less surgical time. ^12^^,^^13^ In some situations, a reverse gracilis muscle flap could be performed. ^14^ The accurate calibration of pedicles and best vasculature of gracilis makes it a best candidate for the distally based pedicle flap. ^3^ Gracilis receives pedicles ranging between one and five and 75% of the limbs have two vascular pedicles. ^15^ In our study, the pedicles ranged between one and three. It was reported that the first vascular pedicle is placed at a distance of 105±20 mm from the pubic tubercle. ^15^ In the present study, this distance was 93.6±35.6 mm on the right lower extremity and left lower extremity had still proximal topography of the first pedicle. In another study, ^16^ it was reported that the proximal and distal pedicles of the gracilis were located 60 mm and 266 mm away from the pubic tubercle. We observed the distal pedicle at 214.6±86.8 mm and 145.3±124.4 mm away over the right and left sides. In another study, the most proximal pedicle entered the gracilis at a distance of 160-180 mm. Topographic data of vascular pedicles have implications in the field of plastic and reconstructive surgeries. In this concept, this study offers a detailed topographic anatomy of the pedicles in the gracilis muscle.

This study also provided the length and width of the gracilis; however, thickness was not measured. This can be considered as a limitation of the present study. Age- and sex-based segregation were not possible. This study had a smaller sample size, and the data were better interpreted based on collections from a larger sample size. The observed length of gracilis in this study was 369.9±34.1 mm and 359.6±29.6 mm over the right and left sides. This is smaller than previously published data, which reported it to be 410±21 mm, ^1^ the difference is due to ancestral variations. Caucasians were tall and robust, and our samples had smaller dimensions. In another study, the length of gracilis was 432±20.8 mm in males and 371±7.6 mm in females. In their study, the distance of pedicle was 94±7.2 mm in males and 79±2.6 mm in females. ^17^ We could not segregate the specimens into males and females in this study and this assessment was not possible.

## Conclusion

The present study provided morphometric data ^18^ of the gracilis and the topographical anatomy of the vascular pedicles. These data will assist surgeons in plastic and reconstructive surgery.

### Ethical consideration

The institutional ethics committee, Kasturba Medical College, Mangalore (Reg. No. ECR/541/Inst/KA/2014/RR-17) has reviewed the protocol of this study and approved on seventeenth of October, 2018 (IEC KMC MLR 10-18/395).

## Data Availability

Figshare: Medline database search strategy for ‘Morphometry of gracilis’ Figshare: Medline database search strategy for ‘Morphometry of gracilis. figshare. Dataset, https://doi.org/10.6084/m9.figshare.24503989.v1’. ^18^ The project contains the following underlying data (file name – Morphometry of … Gracilis). Data are available under the terms of the Creative Commons Zero “No rights reserved” data waiver (CC0 1.0 Public domain dedication).
