# Supplementary material for: Morphology of gracilis muscle and the topographic anatomy of its neurovascular pedicles: A cross sectional study
Source: F1000Res. 2025 Nov 12;13:299. Originally published 2024 Apr 18. [Version 2] doi: 10.12688/f1000research.144786.2 (PMC12749558; doi:10.12688/f1000research.144786.2)
Supplement: Peer review history

# Reviewer report: Reviewer response for version 2

**DOI:** 10.5256/f1000research.190994.r432307

**Published:** 2025-12-29

**Authors:** Nagavalli Pushpa

**Affiliations:** 1 Department of Anatomy, JSS Medical College, JSS Academy of Higher Education and Research, Mysore, Karnataka, India

Dear Author,

 In the introduction please add why Gracilis is the choice for graft. 

 Please provide reference for the methodology. Mention how did you arrive at the present sample size. 

 Kindly add some more points in the discussion and make it robust.

 Manuscript is an useful study indicating the morphometry of the Gracilis.

Is the work clearly and accurately presented and does it cite the current literature?

Yes

If applicable, is the statistical analysis and its interpretation appropriate?

No

Are all the source data underlying the results available to ensure full reproducibility?

Yes

Is the study design appropriate and is the work technically sound?

Yes

Are the conclusions drawn adequately supported by the results?

Yes

Are sufficient details of methods and analysis provided to allow replication by others?

Yes

Reviewer Expertise:

Gross anatomy, Histology, Neuroanatomy and embryology.

I confirm that I have read this submission and believe that I have an appropriate level of expertise to confirm that it is of an acceptable scientific standard.

# Reviewer report: Reviewer response for version 2

**DOI:** 10.5256/f1000research.190994.r432309

**Published:** 2025-12-26

**Authors:** Apurba Patra

**Affiliations:** 1 All India Institute of Medical Sciences, Bathinda, Punjab, India

The present artile describing thet morphology of gracilis muscle and the topographic anatomy of its neurovascular pedicles: A cross sectional study is very well executed and well written. This work bridges the gap in the existing literature regrading the morphology and topographic location of gracilis muscle, commonly used as a graft.

Is the work clearly and accurately presented and does it cite the current literature?

Yes

If applicable, is the statistical analysis and its interpretation appropriate?

Yes

Are all the source data underlying the results available to ensure full reproducibility?

Yes

Is the study design appropriate and is the work technically sound?

Yes

Are the conclusions drawn adequately supported by the results?

Yes

Are sufficient details of methods and analysis provided to allow replication by others?

Yes

Reviewer Expertise:

Translational research in Morphology, Neuroanatomy

I confirm that I have read this submission and believe that I have an appropriate level of expertise to confirm that it is of an acceptable scientific standard.
